# Massive Diversity in Whole-Genome Sequences of Streptococcus suis Strains from Infected Pigs in Switzerland

**DOI:** 10.1128/MRA.01656-18

**Published:** 2019-01-31

**Authors:** Marc J. A. Stevens, Nathalie Spoerry Serrano, Nicole Cernela, Sarah Schmitt, Jacques Schrenzel, Roger Stephan

**Affiliations:** a Institute for Food Safety and Hygiene, Vetsuisse Faculty, University of Zurich, Zurich, Switzerland; b Section of Veterinary Bacteriology, Vetsuisse Faculty, University of Zurich, Zurich, Switzerland; c Bacteriology Laboratory, Geneva University Hospitals and University of Geneva, Geneva, Switzerland; University of Maryland School of Medicine

## Abstract

Here we report the whole-genome sequences of 15 clinical Streptococcus suis strains isolated from pigs in Switzerland. Although they originated from the same host and geographic origin, the strains showed a large amount of diversity.

## ANNOUNCEMENT

Streptococcus suis is a Gram-positive, facultative, anaerobic bacterium that is mainly found in the nasal mucosa and the tonsils of healthy pigs. Under predisposing circumstances, like inadequate sanitation or reduced immunity, S. suis can cause various diseases such as meningitis, septicemia, arthritis, pneumonia, and endocarditis (1). Besides being an important pig pathogen causing major economic losses, S. suis is considered a relevant zoonotic agent, especially in China and Southeast Asia (1–3). Currently, there are 29 described S. suis serotypes. Worldwide, serotype 2 is the most common reported serotype to cause infections in pigs, followed by serotypes 9 and 3 (1). In humans, the most frequently identified serotypes are serotype 2, followed by serotype 14 (1).

We have sequenced 15 clinical S. suis strains (Table 1). The strains were originally isolated between 2006 and 2018 by streaking pig samples onto Columbia agar with sheep blood (Thermo Fisher Diagnostics AG, Pratteln, Switzerland). The plates were incubated at 37°C for 48 hours under aerobic conditions. Strains were identified by matrix-assisted laser desorption ionization–time of flight mass spectrometry (MALDI-TOF MS) (Biotyper Compass Explorer software v.4.1.60, Bruker Daltonics, Bremen, Germany) and serotyped by multiplex PCR according to Kerdsin et al. (4).

**TABLE 1:** Overview of strains

Strain	Serotype	Yr	Source	GenBank accession no.	SRA accession no.	No. of contigs	Genome size (bp)	*N*_50_	GC content (%)
PP203	9	2015	Blood from heart	RSDR00000000	SRR8290481	27	2,127,065	6	41.4
PP269	1 or 14	2015	Blood from heart	RSDQ00000000	SRR8290480	63	1,968,146	12	41.3
PP386	6	2016	Blood from heart	RSDP00000000	SRR8290479	53	1,878,848	12	41.5
PP422	9	2016	Lung	RSDO00000000	SRR8290478	48	2,075,657	8	43.7
PP423	2 or 1/2	2016	Blood from heart	RSDN00000000	SRR8290477	51	2,068,343	8	41.2
PP425	6	2016	Brain	RSDM00000000	SRR8290476	54	1,881,239	11	41.5
PP463	2 or 1/2	2016	Blood from heart	RSDL00000000	SRR8290475	52	2,135,450	9	41.1
PP464	ND^*a*^	2016	Lung	RSDK00000000	SRR8290474	94	2,340,449	15	41.5
PP536	9	2016	Heart	RSDJ00000000	SRR8290473	24	2,122,156	4	41.4
PP730	1 or 14	2018	Joint	RSDI00000000	SRR8290472	58	1,912,461	11	41.4
PP735	1 or 14	2018	Joint	RSDH00000000	SRR8290484	59	1,912,627	11	41.4
SS1014	ND	2010	Kidney	RSDG00000000	SRR8290483	208	2,504,491	37	41.2
SS29	6	2006	No information	RSDF00000000	SRR8290486	49	1,894,451	11	41.5
SS470	2 or 1/2	2007	Heart	RSDE00000000	SRR8290485	49	2,079,888	8	41.1
SS8	6	2006	No information	RSDD00000000	SRR8290482	48	1,893,520	11	41.5

a ND, not determined.

Genomic DNA was extracted using a DNA blood and tissue kit (Qiagen, Hombrechtikon, Switzerland) and prepared for sequencing with a Nextera DNA Flex sample preparation kit (Illumina, San Diego, CA, USA) on an Illumina MiniSeq sequencer with 150-bp paired-end reads.

The sequencing resulted in an output of paired-end read sets containing 596,559 to 1,666,705 reads of 150 bp. The quality of the reads was checked using FastQC (https://www.bioinformatics.babraham.ac.uk/projects/fastqc/). The reads passed all quality steps with the exception of the control step “per-base sequence content.” Failure to pass this step, however, is typical for transposon-based libraries (FastQC manual [see https://www.bioinformatics.babraham.ac.uk/projects/fastqc/]) and was ignored. Reads were assembled *de novo* using Spades 3.12 (5) with activation of the “–careful” option. Raw assemblies were filtered for size larger than 1,000 bp and coverage of more than 25-fold.

The final assemblies resulted in 13 genomes with a size between 1,878,848 and 2,135,450 bp and coverages between 50- and 120-fold (Table 1). The genomes consisted of 24 to 64 contigs per strain, and the largest contigs were 129 to 333 kbp. Strains SS1014 and PP464 had large genomes of 2,340,449 and 2,504,491 bp, with coverages of 50- and 39-fold, respectively. The SS1014 and PP464 genomes consisted of 204 and 94 contigs, the largest of which were 76 kb and 174 kb, respectively.

The average nucleotide identity (ANI) of the strains was calculated according to Richter et al. (6) using PyANI (https://github.com/widdowquinn/pyani). The ANI of strain PP422 was only 88 to 89% compared to the other strains in this study, thus showing a high genomic diversity which was already observed previously in this species (7). The other strains had an ANI of at least 94.6%, which is below the ANI cutoff for species differentiation of 95 to 96% (6), confirming again the diversity of the species S. suis.

Our results highlight the massive diversity within the pathogenic species S. suis, even between strains from the same host and region. Since the breeding of pigs is quite consolidated in Switzerland, this opens up possibilities for strain tracing in case of human disease outbreaks.

### Data availability.

All these sequences have been published in GenBank under SRA accession no. SRR8290481 (PP203), SRR8290480 (PP269), SRR8290479 (PP386), SRR8290478 (PP422), SRR8290477 (PP423), SRR8290476 (PP425), SRR8290475 (PP463), SRR8290474 (PP464), SRR8290473 (PP536), SRR8290472 (PP730), SRR8290484 (PP735), SRR8290483 (SS1014), SRR8290486 (SS29), SRR8290485 (SS470), and SRR8290482 (SS8). All these sequences have also been published in GenBank under the genome accession no. RSDR00000000 (PP203), RSDQ00000000 (PP269), RSDP00000000 (PP386), RSDO00000000 (PP422), RSDN00000000 (PP423), RSDM00000000 (PP425), RSDL00000000 (PP463), RSDK00000000 (PP464), RSDJ00000000 (PP536), RSDI00000000 (PP730), RSDH00000000 (PP735), RSDG00000000 (SS1014), RSDF00000000 (SS29), RSDE00000000 (SS470), and RSDD00000000 (SS8).
